# The Spectrum of Extraglandular Manifestations in Primary Sjögren’s Syndrome

**DOI:** 10.3390/jpm13060961

**Published:** 2023-06-07

**Authors:** Ancuta Mihai, Constantin Caruntu, Ciprian Jurcut, Florin Cristian Blajut, Mihnea Casian, Daniela Opris-Belinski, Ruxandra Ionescu, Ana Caruntu

**Affiliations:** 1 Department of Internal Medicine, Carol Davila Central Military Emergency Hospital, 010825 Bucharest, Romania; coca.ancuta@gmail.com (A.M.); c.jurcut@gmail.com (C.J.); 2 Department of Rheumatology, Faculty of General Medicine, Titu Maiorescu University, 031593 Bucharest, Romania; 3 Department of Physiology, Carol Davila University of Medicine and Pharmacy, 020021 Bucharest, Romania; costin.caruntu@gmail.com; 4 Department of Dermatology, Prof. N.C. Paulescu National Institute of Diabetes, Nutrition and Metabolic Diseases, 011233 Bucharest, Romania; 5 Department of General Surgery, Carol Davila Central Military Emergency Hospital, 010825 Bucharest, Romania; cristi.blajut@gmail.com; 6 Department of Medical-Surgical Specialties, “Titu Maiorescu” University of Bucharest, 040441 Bucharest, Romania; 7 Emergency Institute for Cardiovascular Diseases Prof. Dr. C.C. Iliescu, 022328 Bucharest, Romania; mihnea.casian@gmail.com; 8 Department of Cardiology, Carol Davila University of Medicine and Pharmacy, 020021 Bucharest, Romania; 9 Internal Medicine and Rheumatology Department, Sfanta Maria Clinical Hospital, 011172 Bucharest, Romania; danaopris0103@yahoo.com (D.O.-B.); ruxandraionescu1@gmail.com (R.I.); 10 Internal Medicine and Rheumatology Department, Carol Davila University of Medicine and Pharmacy, 020021 Bucharest, Romania; 11 Department of Oral and Maxillofacial Surgery, Carol Davila Central Military Emergency Hospital, 010825 Bucharest, Romania; 12 Department of Oral and Maxillofacial Surgery, Faculty of Dental Medicine, Titu Maiorescu University, 031593 Bucharest, Romania

**Keywords:** Primary Sjögren Syndrome, extraglandular manifestations, pathophysiology, diagnosis, treatment, future perspectives

## Abstract

Extraglandular manifestations (EGMs) in primary Sjogren’s syndrome (pSS) represent the clinical expression of the systemic involvement in this disease. EGMs are characterized by a wide heterogeneity; virtually any organ or system can be affected, with various degrees of dysfunction. The existing gaps of knowledge in this complex domain of extraglandular extension in pSS need to be overcome in order to increase the diagnostic accuracy of EGMs in pSS. The timely identification of EGMs, as early as from subclinical stages, can be facilitated using highly specific biomarkers, thus preventing decompensated disease and severe complications. To date, there is no general consensus on the diagnostic criteria for the wide range of extraglandular involvement in pSS, which associates important underdiagnosing of EGMs, subsequent undertreatment and progression to severe organ dysfunction in these patients. This review article presents the most recent basic and clinical science research conducted to investigate pathogenic mechanisms leading to EGMs in pSS patients. In addition, it presents the current diagnostic and treatment recommendations and the trends for future therapeutic strategies based on personalized treatment, as well as the latest research in the field of diagnostic and prognostic biomarkers for extraglandular involvement in pSS.

## 1. Introduction

**Figure 1:**
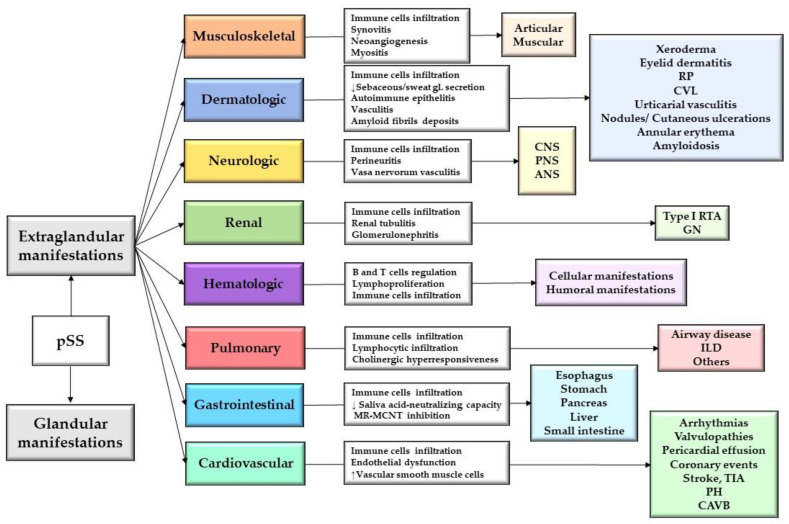
Extraglandular manifestations in pSS. Abbreviation: RP, Raynaud phenomenon; CVL, cutaneous vasculitis lesions; CNS, central nervous system; PNS, peripheral nervous system; ANS, autonomic nervous system; RTA, renal tubular acidosis; GN, glomerulonephritis; ILD, interstitial lung disease; MR- MCNT, inhibit muscarinic receptor-mediated cholinergic neurotransmission; TIA, transient ischemic attacks; PH, pulmonary hypertension; CAVB, congenital atrioventricular block. ↓, low; ↑, high.

Primary Sjögren Syndrome (pSS) is a systemic chronic autoimmune rheumatic disorder of unknown etiology, characterized by lymphocytic infiltration with immune-mediated destruction of exocrine glands, primarily including salivary and lacrimal glands [1]. The dryness may affect other mucosal surfaces such as the airways, digestive tract, and vagina, leading to the clinical picture of the sicca syndrome [2]. In addition to these, pSS can involve any organ system, expressed in various and complex clinical extraglandular manifestations (EGMs). Extraglandular symptoms are divided into non-visceral, represented by musculoskeletal and cutaneous manifestations, and visceral symptoms, which include neurological, renal, hematological, pulmonary, gastrointestinal, and cardiovascular manifestations (Figure 1) [3]. The clinical expression of pSS is similar to secondary Sjögren Syndrome (sSS), which is characterized by simultaneous association with other autoimmune diseases, such as rheumatoid arthritis (RA), systemic sclerosis (SSc) or systemic lupus erythematosus (SLE), or with concomitant organ-specific autoimmune diseases, such as autoimmune thyroiditis, primary biliary cholangitis (PBC), and autoimmune hepatitis (AIH) [4]. Therefore, the distinction between pSS and sSS is reflected only by the overlap with other autoimmune diseases. The patients’ management is similar in both types of disease [1].

The classification criteria for pSS were published in 2016 by ACR/EULAR (American College of Rheumatology/European League against Rheumatism) [5]. These classification criteria apply to any patient with symptoms of ocular or oral dryness according to American European Consensus Group criteria (AECG) questions or to patients with the positivity of at least one of the domains of the EULAR Sjögren’s syndrome disease activity index (ESSDAI) questionnaire. The pSS diagnostic is considered in subjects that have a total score ≥ 4, obtained of the five items: anti-SSA/Ro antibody positivity, labial salivary gland biopsy with focal lymphocytic sialadenitis and focus score of ≥1 foci/4 mm^2^, abnormal Ocular Staining Score (OSS) of ≥5 (or van Bijsterveld score of ≥4), Schirmer’s test result of ≤5 mm/5 min and an unstimulated salivary flow rate of ≤0.1 mL/min [5].

## 2. Pathogenic Mechanisms of pSS

The interaction between genetic and environmental factors is thought to play a crucial role in susceptible individuals, leading to the dysregulation of the immune system and pSS development [6]. The function of specific cytokines and chemokines, and their expression by cells of the innate and adaptive immune systems are actively involved in pSS pathogenesis, including extraglandular involvement [1,7].

### 2.1. Viral/Infectious Factors

Different infectious agents, especially viruses, have been considered potential pSS pathogenetic triggers [8]. For example, Epstein–Barr virus (EBV) was identified in saliva samples and in salivary and lacrimal gland biopsies of pSS patients with EGMs, particularly with myopathies [9]. Furthermore, EBV has a well-established tropism for B cells, favoring the development of lymphoproliferative processes, the most severe extraglandular complication in pSS [10]. However, to date, no clear association with viral infections, such as human herpes virus-6 (HHV-6), hepatitis C or B viruses, human immunodeficiency virus (HIV), human T-lymphotropic virus type 1 (HTLV1), or Coxsackie A virus, have been reported in pSS [11].

### 2.2. Genetic and Epigenetic Factors

Genetic factors play an important role in pSS pathogenesis [12]. Thus, associations between Human Leukocyte Antigen (HLA) alleles, such as DRB1*03:01, DQA1*05:01, DQB1*02:01, and pSS susceptibility, were identified by genomic studies [13]. Moreover, six non-HLA regions were shown to be involved in pSS, including interferon regulatory factor 5 (IRF5), signal transducer and activator of transcription 4 (STAT4), BLK, interleukin (IL)-12A, TNFAIP3 interacting protein 1 (TNIP1), and C-X-C motif chemokine receptor 5 (CXCR5). The HLA-DQB1*0201 allele and the expression of IRF5 and STAT4 seem to have the strongest association with pSS [14,15].

Furthermore, based on genome-wide studies, key steps in pSS triggering were identified, such as aberrant activation of the innate immune response, through the IFN and NF-kB pathways, atypical recruitment to lymphoid sites, and T-cell activation with HLA susceptibility ascending [16].

### 2.3. Acquired Immunity

T cells significantly contribute to pSS pathogenesis. CD4+ T cells differentiate into the two subtypes, T helper (Th)1 and Th2 cells [1,17]. Th1 cells mainly produce pro-inflammatory cytokines such as IFN-γ and IL-2 [18], while Th2 cells produce anti-inflammatory cytokines such as IL-4, IL-5, IL-9, IL-10, IL-13, and IL-25 [18,19]. Abnormal Th1 activation was detected in the salivary gland infiltrate from pSS patients, accompanied by elevated levels of IFN-γ and Th1 cells in the blood [20]. Th2-related marker transcripts were identified in germinal centers from salivary gland biopsies of pSS patients, alongside an intense B cell infiltration [21]. Th17 cells play a fundamental role in maintaining mucosal barrier integrity by inducing the synthesis of tight junction proteins and playing a defensive role against infections in healthy individuals [22,23,24]. In autoimmune disease, Th17 cells produce IL-17 and other inflammatory cytokines such as TNF-α, IL-22, and IL-26, inducing and mediating pro-inflammatory responses [25]. The affected salivary glands of pSS patients represent a perfect environment for the recruitment of Th17 cells [26]. T-regulatory cells (Tregs), responsible for immune homeostasis, suppression of autoreactive lymphocytes and release of different cytokines, such as IL-10 and TGF-β [27], have been detected with increased values in blood samples from pSS patients with EGMs, while a reduction in Treg cells seems to prevent the emergence of EGMs in these patients [28,29]. Follicular regulatory T cells (Tfr), a subtype of Treg specialized in the regulation and suppression of T helper follicular and B cell activity [30], have been reported in higher numbers in blood and salivary glands analysis of pSS patients [31,32].

B lymphocytes represent one of the hallmarks of pSS, and their dysregulation also plays a key role in autoimmunity processes and extraglandular manifestations, particularly in lymphoma development [33,34]. In pSS patients, the properties of regulatory B cells have been attributed to their ability to secrete cytokines, such as IL-6 and IL-10 [35]. IL-6 is a primary cytokine which plays a pivotal role in promoting the synthesis of autoantibodies through secondary cytokine production by local B lymphocytes [36]. Moreover, IL-6 may synergize with IL-1β and transforming growth factor (TGF) β to modify the polarization of Th cells into Th17 [37]. In experimental studies, IL-6 deficiency may reduce autoantibody production and the subsequent inflammation in specific disease organs [38]. IL-10-producing regulatory B (Breg) cells play a critical role in maintaining immune tolerance in inflammatory reactions. IL-10, a pleiotropic and immunoregulatory cytokine, contributes to the delicate balance between inflammation and immunoregulation [39]. This anti-inflammatory cytokine may diminish the production of pro-inflammatory cytokines and chemokines, including IL-1β, IL-6, IL-8, TNF-α, and IL-12, controlling organ-specific inflammation [40]. Furthermore, in salivary glands, germinal centers have been identified, sites able to promote chronic activation of B lymphocytes followed by lymphoma development in pSS patients [41]. B cell receptor CXCR5 seems to be an important element in the formation of germinal centers in pSS with extraglandular involvement [42]. The association between B cell lymphomas and pSS will be further detailed in the hematologic manifestations section of the review.

B cell activating factor (BAFF, also known as BLyS) is produced by monocytes, macrophages, and dendritic cells (DCs), and is part of the TNF family, playing a vital role in B cell survival [43]. In pSS patients, salivary epithelial cells, T and B cells can produce BAFF [44]. Additionally, type I and II IFNs were shown to induce BAFF production [45]. In pSS patients, BAFF levels are significantly increased, not only in the blood, where they correlate with the levels of anti-Ro/SSA and anti-La/SSB antibodies and disease activity but also in the salivary glands [46]. Patients with high BAFF levels have a more pronounced B cell activation that leads to lymphoid proliferations in pSS patients with EGMs [47].

### 2.4. Innate Immunity

In pSS, DCs present an aberrant phenotype leading to their accumulation in the salivary glands. In saliva specimens from pSS patients, an upregulation of chemokines receptors and ligands was detected, with impact on the effective migration of DCs to inflamed tissues [1,48]. On the other hand, lower numbers of circulating DCs reported in pSS patients may be secondary to the aberrant regulation of apoptosis [48]. DCs can be activated by self-antigens, Toll-like receptor (TLR) type, leading to the production of type I interferon (IFN) [49]. Furthermore, IFN induces the production of the BAFF by monocyte circulating cells, a scenario in which the DCs contribute to the activation and differentiation of B cells into plasma cells, resulting in the secretion of antibodies [50]. These mechanisms could also influence the development of lymphoma, the most feared EGM in pSS [51].

Epithelial cells are considered major players in the pathogenesis of pSS, representing the target of the autoimmune process and also the triggers of immune activation [52]. They also regulate the processes of the expression of ribonucleoprotein complexes, Ro/SSA, and La/SSB, secondary to apoptotic mechanisms [53]. In addition to the fact that they modulate the production of cytokines, such as BAFF, epithelial cells also regulate the expression of chemokines, responsible for the recruitment of leukocytes [54,55]. Furthermore, through the expression of costimulatory proteins on their surface, epithelial cells control the interaction with T cell population [31]. In pSS, local inflammation and production of proinflammatory cytokines, such as IFN-gamma and tumor necrosis factor (TNF)-alpha, lead to the disruption of the tight junction between epithelial cells, which secondarily contributes to the emergence of both glandular and extraglandular dysfunctions in pSS patients [56,57].

Natural Killer (NK) cells have also been incriminated in pSS pathogenesis [58]. In the salivary glands of pSS patients, subsets of unconventional NK cells that may produce inflammatory cytokines such as IL-22 were identified [59]. In addition, NK cells can facilitate BAFF production [60]. Furthermore, in pSS patients with EGMs, a higher number of NK cells was detected, implying their role in clinically aggressive disease [61].

Transcriptional analyses yielded an overexpression of IFN-inducible genes, also known as type I and II IFN-signature, which were identified in peripheral blood mononuclear cells and salivary gland tissue specimens from pSS patients [62]. Both IFNs signatures demonstrated their association with the development of EGMs in pSS [63]. Particularly, the type II IFN signature seems to be associated with a higher risk for lymphoma development [64]. A bidirectional interaction between the IFN pathway and B lymphocyte activation was suggested [65]. Therefore, IFN induces B cell hyperresponsiveness, which in turn favor the production of autoantibodies [66]. The significant roles of BAFF and IFN signatures in pSS pathogenesis could represent an opportunity for novel therapeutic targets in pSS [43].

## 3. Extraglandular Manifestations

During disease progression, most pSS patients will develop EGMs [12]. The severity and type of symptoms can vary widely from one patient to another and can significantly impact their quality of life [67]. The effective management of EGMs implies an early diagnosis, if possible before their clinical expression by means of predictive biomarkers, efficient and accurate investigation tools and scores, as well as personalized treatments, aiming to prevent complications and improve the patient’s quality of life. In current clinical practice, the wide panel of extraglandular manifestations found in pSS patients is included in the ESSDAI scoring system [68].

### 3.1. Musculoskeletal Manifestations

Musculoskeletal involvement is present in the majority of pSS patients, consisting of arthralgias, arthritis, and myalgias [69]. The prevalence of arthralgia is up to 96% [70], whereas arthritis has been reported in 16.6% [71]. Arthralgias may appear concurrently with the sicca symptoms and correlate with anti-Ro/SSA anti-La/SSB antibodies positivity [72]. Arthritis was reported to be intermittent, predominantly polyarticular, symmetric, and non-destructive, occasionally involving the proximal interphalangeal and metacarpophalangeal joints and wrists [73,74]. Fewer cases of monoarthritis and axial involvement have been reported [75]. Over 70% of pSS patients may complain of myalgias [76], while active myositis was reported in 0.85–14% [77]. Within the broad myositis spectrum, the inclusion body myositis (IBM), a late complication of pSS, was present in 0.5% of pSS patients [77,78]. The coexistence of myositis-specific antibodies, such as anti cytosolic 5′-nucleotidase 1A (NT5c1A) antibodies, with anti-SSA/Ro antibodies, is reported in up to 12% of pSS patients [79].

#### 3.1.1. Pathophysiology

Synovitis can be present in joint involvement, described as inflamed synovial tissue hyperplasia of the intimal lining layer, due to the accumulation of macrophages and proliferation of fibroblast-like synoviocytes [80]. Furthermore, neoangiogenesis with endothelial activation in the synovial tissue and accumulation of inflammatory cells, such as macrophages, DCs, lymphocytes, and mast cells, under the synovial lining have been reported [81]. These infiltrating cells are activated and produce a wide range of pro-inflammatory mediators that contribute to synovitis and lead, in some patients, to cartilage and bone destruction [82]. In muscular involvement, the deregulation of autophagy and anti-cN1A auto-immunity in HLA-DR3 genetic background were observed [77]. The main finding on muscle biopsy samples seems to be a perivascular lymphocytic inflammatory infiltrate, composed of CD 4+ T lymphocytes and B-cells [83]. Nonspecific myositis, vasculitis, and necrotic lesions were reported in isolated cases [76].

#### 3.1.2. Diagnosis

In musculoskeletal manifestations, the diagnosis is mainly clinical, and can be completed with laboratory and imaging investigations. In addition to standard X-rays, the musculoskeletal ultrasound sonography with power Doppler can be useful, especially in active arthritis [84]. In complex cases, magnetic resonance imaging (MRI) is recommended. In muscular involvement, muscular weakness, high values in creatinine phosphokinase (CPK), and an abnormal electromyogram (EMG) can be observed, and further muscle biopsy with immunohistochemical staining can be performed, as the gold standard for the diagnosis [83].

#### 3.1.3. Perspectives for Therapeutic Management of Musculoskeletal Manifestations in pSS

The recommended treatment in mild and intermittent inflammatory joint pain may consist of non-steroidal anti-inflammatory drugs (NSAIDs), while, in acute flares, intra-articular, intra-muscular, or oral steroids may be an option, allowing an immediate response [85]. For most patients with joint symptoms, hydroxychloroquine (HCQ) is the next treatment option [86]. Even though the studies using HCQ in pSS are inconclusive, this approach is recommended in the EULAR guidelines for the management of pSS [85]. Methotrexate (MTX) is strongly suggested as an alternative treatment for arthritis, either alone or in addition to HCQ [85,87]. In refractory cases to HCQ and/or MTX, alternative options, such as corticosteroids, leflunomide, sulfasalazine, azathioprine (AZA), cyclosporine A, or biologic drugs may be considered [88]. Anti-TNF-α and anti-CD-20 drugs have demonstrated a limited benefit in the control of systemic manifestations, while the CTLA fusion protein (CTLA4), abatacept, seems to bring hope in the alleviation of musculoskeletal manifestations of pSS patients [89,90,91]. Anti-BLyS/BAFF therapy, belimumab, seems to improve arthritis manifestations in pSS patients [92], while ianalumab (VAY736), another B cell-depleting BAFF-R blocker, provided promising results in recent studies [93]. However, to date, no biologic drugs have been approved by the regulatory agencies, the Food and Drug Administration (FDA) or the European Medicines Agency (EMA), for the treatment of pSS. Their use is experimental/investigational in ongoing clinical trials (Table 1).

### 3.2. Dermatological Manifestations

A large spectrum of skin manifestations may be present in patients with pSS, from common xeroderma to severe vasculitis, including other rare associated conditions [110]. The incidence of cutaneous manifestations in pSS has a female predominance, with a prevalence of up to 72%, making skin involvement one of the most common EGMs in pSS (Table 2) [111,112]. Xeroderma is the most typical cutaneous manifestation of pSS, with a prevalence of up to 72% [112]. Similar to xerosis, eyelid dermatitis has been reported in 42% of pSS patients as a lichenification or thickening of the skin, along with erythema, pigmentation, or papules [111,113]. Another cutaneous manifestation is Raynaud’s phenomenon (RP), with a prevalence between 16–35% in pSS patients [114].

Cutaneous vasculitis lesions (CVL) can be observed in pSS patients, manifested as palpable purpura and non-palpable purpura [115,116]. CVL is considered the most clinically and prognostically significant cutaneous complication of pSS patients [110]. The prevalence of CVL has been reported in 10–30% of pSS patients [111,117], while its clinical manifestation, palpable purpura, was found in 80–90% of CVL [118]. Therefore, CVL are frequently associated with other systemic manifestations, more severe disease, lymphoma, and poor prognosis, especially when serum cryoglobulins are present [115,119]. Patients with vasculitis have a higher prevalence of anti-Ro/SSA and/or anti-La/SSB antibodies, and about one-third of them have positive cryoglobulins [111]. Alongside the CVL, cutaneous ulcers, urticarial vasculitis, or skin nodules may appear in pSS patients [116,120].

Cutaneous amyloidosis is rarely found in pSS, and can manifest as a single nodule, or sometimes multiple nodules, mainly on the legs, arms, trunk, and face [115]. Localized amyloid light-chain (AL) amyloidosis can occur in the skin, lungs, eyes, and bladder of pSS patients [121,122], while cutaneous amyloid A (AA) amyloidosis, is uncommon in pSS and has been reported in association with celiac disease [123]. Annular erythema (AE), an erythematous non-scarring dermatosis, characterized by a wide elevated border and a central pallor area, is commonly found in Asian patients [124], compared with only 9% in non-Asian populations [125]. In subacute cutaneous lupus (scLE), an entity similar to AE, studies have reported a strong association with the positivity for anti-Ro/SS-A and/or anti-La/SS-B autoantibodies [8,111].

**Table 2:** The most frequent cutaneous manifestations in pSS.

Cutaneous Manifestations	% pSS Patients
Xeroderma	72% [110]
Eyelid dermatitis	42% [113]
Raynaud phenomenon	16–35% [114]
Cutaneous vasculitis lesions	10–30% [117]
Urticarial vasculitis	0.8–21% [120]
Annular erythema	9% [125]

Less common dermatologic manifestations noted in pSS include pruritus, vitiligo, alopecia, anetoderma, Sweet syndrome, lichen planus, granulomatous panniculitis, subcorneal pustular dermatosis, erythema elevatum diutinum, erythema multiforme-like, erythema perstans-like, erythema nodosum-like lesions, lymphomatoid papulosis, and cutaneous T-cell lymphoma [115].

#### 3.2.1. Pathophysiology

In xeroderma, an alteration in the protective function of the outer layer of the skin was described, with decreased sebaceous and sweat gland secretion, and the so called autoimmune epithelitis, defined as an increased infiltration with autoreactive T and B cells [126,127]. It also involves circulating immune complexes, complement activation, cytokine production, as well as endothelial cell damage, resulting in the loss of their fibrinolytic properties, with fibrin deposition and degeneration of affected vessels [128]. Eyelid dermatitis etiology is attributed to chronic mechanical trauma through the rubbing of the periorbital area. The histopathologic examination revealed interface dermatitis, multiple melanophages, and a dense lymphocytic infiltration around hair follicles [129]. In vasculitis, the most common finding on pathology specimens, aspects of leukocytoclastic vasculitis followed by cryoglobulinemic and urticarial vasculitis were described [115,130]. This leukocytoclastic vasculitis is characterized by the fibrinoid necrosis of the vessel walls, leukocytosis and extravasation of erythrocytes, and the presence of IgM, IgG, and C3 around the vessel [111,131]. In urticarial vasculitis, lesions arise due to the activation of mast cells, which release histamine, resulting in vasodilatation, increased vascular permeability, and dermal oedema [111]. Amyloidosis is characterized by the extracellular deposition and accumulation of amyloid fibrils, AL or AA proteins, that can be objectified in green fluorescence under polarized light microscopy or by Congo red staining of tissue samples [121]. For annular erythema/scLE, the histology exam showed perivascular and periadnexial lymphocytic infiltration with dermal mucin deposits [125].

#### 3.2.2. Diagnosis

**Figure 2:**
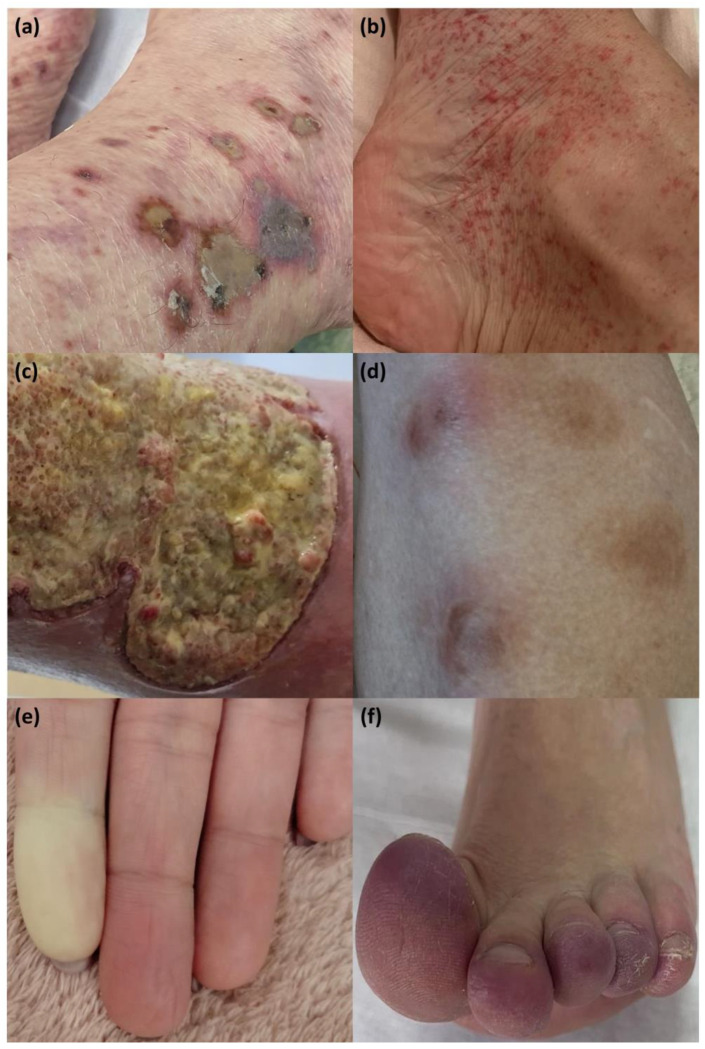
Cutaneous manifestations in pSS patients. (**a**) Palpable purpura; (**b**) Non-palpable purpura; (**c**) Cutaneous ulcers in vasculitis; (**d**) Erythema nodosum-like lesions; (**e**) Raynaud syndrome sign; (**f**) Chronic chilblains.

The diagnosis of cutaneous manifestations is mostly clinical (Figure 2), while skin biopsy is recommended in complex cases. Recent clinical studies have identified biological elements, such as monocytes to lymphocyte ratio (NLR), platelet to lymphocyte ratio (PLR), monocytes to lymphocyte ratio (MLR), or gammaglobulins as predictive parameters for cutaneous involvement in pSS patients [116,132].

#### 3.2.3. Treatment of Cutaneous Manifestations in pSS

Treatment of cutaneous manifestations in pSS patients varies from local emollients in the xeroderma to systemic immunosuppression in CVL. In cutaneous vasculitis, the treatment choice depends on the extent and degree of the manifestation and requires glucocorticoids (GCs) with or without systemic immunosuppression, such as AZA or MTX [133]. In refractory cases, cyclophosphamide (CYC) is most commonly prescribed, while in cryoglobulinemic vasculitis, administration of rituximab, or belimumab, provided promising results [134,135]. Annular erythema has a slow response to topical therapy, while GCs, calcineurin inhibitors, and hydroxychloroquine (HCQ) showed a good perspective [124]. The therapy for localized cutaneous amyloidosis is challenging and involves cryotherapy, electrodissection and curettage, intralesional triamcinolone injections, or ablative laser therapy [136].

### 3.3. Neurologic Manifestations

Neurologic manifestations may involve the central nervous system (CNS), with a prevalence of around 5% [137], or the peripheral nervous system (PNS), with an incidence between 3.7% to 16% in pSS patients (Table 3) [138,139,140]. CNS involvement in pSS varies from mild cognitive dysfunction to transverse myelitis and paralysis [141]. CNS involvement can include demyelinating diseases, such as neuromyelitis optica, optic neuritis, multiple sclerosis-like disorders, transverse myelitis, lymphocytic meningitis, and possible cerebral vasculitis [142]. Demyelinating CNS lesions can occur in the white matter of the brain and spinal cord of patients with pSS, with an incidence of 3.6–68% [142]. It can mimic the primary progressive forms of multiple sclerosis, with various symptoms, including visual loss, paresis of limbs, ataxia, sphincter dysfunction, cognitive dysfunction, and sensory symptoms [141]. Cranial neuropathy may be present, usually as unilateral pure sensory trigeminal neuralgia that affects the maxillary branch of the trigeminal nerve [141]. Patients with pSS may also present facial nerve neuropathy, and cochlear nerve damage, with both hearing loss and vestibular symptoms [143].

PNS manifestations in pSS patients have various clinical aspects, from axonal sensory and sensorimotor polyneuropathies, small fiber sensory neuropathy, sensory ataxic neuronopathy, cranial nerve neuropathies, radiculoneuropathy, mononeuropathy multiplex, autonomic neuropathies, to chronic inflammatory demyelinating polyneuropathy [141,144]. The most common patterns are pure sensory polyneuropathies and sensorimotor neuropathies, with a prevalence between 40 to 49% and 28 to 56.45%, respectively [140,141]. Distal sensory polyneuropathy affects large nerve fibers and can present indolent and mild paresthesia of the extremities, while sensorimotor polyneuropathy occurs when there is weakness at the same time [139,140]. Mononeuritis multiplex is a painful condition in which damage to two or more nerves occurs in succession leading to sensory and motor deficits [145]. It can be associated with pSS cryoglobulinemic vasculitis and with active systemic disease [142].

**Table 3:** The prevalence of the main neurologic manifestations.

Neurologic Manifestations	% pSS Patients
Central nervous system involvement	5% [137]
Demyelinating lesions	3.6–68% * [142]
Cranial neuropathy	16–20% * [141]
Cognitive dysfunction	53% * [141]
Peripheral nervous system involvement	3.7–16% [138,140]
Pure sensory neuropathy	40–49% ** [138,140]
Sensorimotor polyneuropathies	28–56.45% ** [138,140]
Autonomic nervous system involvement	3–50% [146]

* from patients with central nervous system involvement. ** from patients with peripheral nervous system involvement.

Autonomic nervous system dysfunction was also reported in pSS patients and can manifest as excessive postural tachycardia, orthostatic hypotension, bladder dysfunction, gastrointestinal dysmotility, tonic pupil, segmental hypohidrosis, and diminished sweating [146,147].

#### 3.3.1. Pathophysiology

Different pathogenic mechanisms have been suggested based on the histological and serological findings in pSS patients with neurologic involvement. Vasculitis of the vasa nervorum was described, with lymphocytic, macrophage, and T cell infiltration, as well as necrotizing vasculitis and anti-neuronal antibodies, according to the type of nerve involved [139]. Moreover, perineurial infiltration was observed on nerve biopsies of patients with sensorimotor neuropathy [148]. While in mononeuritis multiplex, inflammation of epineural and perineural blood vessels that perfuse the involved nerves leads to infarction [149] in autonomic nervous system involvement, potential etiologies include cholinergic neurotransmission blockade by cytokines or autoantibodies, T-cell infiltration, and autonomic nerve destruction [141].

#### 3.3.2. Diagnosis

Clinical neurologic symptoms and signs, electromyographic results, and nerve biopsy are the main elements in the diagnosis of peripheric neuropathy [150]. Pseudo blocks corresponding to the areas of nerve ischemia are the distinctive sign detected through electrophysiologic investigation [151]. Skin biopsies revealed a reduced density of epidermal nerve fibers in these patients [152]. In sensory ataxic neuronopathy, magnetic resonance imaging (MRI) may show increased hyperintensity of T2-weighted images in the posterior columns [144]. For demyelinating CNS lesions, MRI may describe a rare form of widespread inflammation of the spinal cord causing T2 hyperintensity, extending across three or more vertebral segments, an aspect suggestive of longitudinally extensive transverse myelitis [153]. The examination of cerebrospinal fluid is mandatory, especially in differential diagnosis with other pathologies [154]. In cognitive dysfunction, neuropsychological tests are the most helpful tools to characterize the nature and degree of impairment [155]. Autonomic testing includes the quantitative sudomotor tests of axonal reflexes and intestinal motility tests for the esophagus, stomach, and small and large bowel [156]. In contrast, the cardiovagal function may be assessed through the measurement of heart rate variability and blood pressure responses with the Valsalva maneuver or tilt-table testing [147]. Cryoglobulinemia, NLR, MLR, gammaglobulins, C4 or vitamin D were suggested in recent studies as predictive markers for neurological involvement in pSS patients [157,158].

#### 3.3.3. Treatment Perspectives in Neurologic Involvement in pSS Patients

As first-line treatment for neuropathic pain, tricyclic antidepressants (TCAs), such as clomipramine and imipramine, and also the serotonin-norepinephrine reuptake inhibitors (SNRIs), such as duloxetine and venlafaxine, have demonstrated their effectiveness [159]. Antiepileptic drugs, such as gabapentin and pregabalin, helped in neuropathic pain alleviation [160,161]. In patients with progressive or refractory symptoms of axonal sensory and sensorimotor neuropathies, oral GCs, intravenous immunoglobulins (IVIg), or mycophenolate mofetil (MMF) are recommended [87,142]. Furthermore, pulse therapy with GCs should be initiated rapidly in vasculitis neuropathies, followed by the addition of CYC [162]. If patients develop CYC toxicity or as maintenance therapy, AZA, and MTX can be used [144,145]. Rituximab, a monoclonal antibody targeting CD20 on B lymphocytes, and long-term anti-BLyS/BAFF therapy, belimumab, revealed promising results in neuroimmune abnormalities in pSS [142,163] (Table 1).

### 3.4. Renal Manifestations

Clinical renal disease is unusual in pSS, being reported in 5% of patients, but it is probably underestimated [8,111,164]. Chronic tubulointerstitial nephritis is the predominant form of pSS-associated renal involvement, which clinically translates mostly into distal renal tubular acidosis (RTA). Furthermore, Type I distal RTA is characterized by a cortical collecting duct dysfunction leading to an impaired H+ elimination [164]. Secondary to tubular defects, patients develop systemic metabolic acidosis or the inability to acidify urine following an oral acid intake [164,165]. Weakness or paralysis due to hypokalemia, renal calculi, or osteomalacia may be present in pSS patients with distal RTA [166,167]. Other dysfunctions involving the cortical collecting duct, the proximal tubular loop of Henle, and the distal convoluted tubule have been reported [167]. Glomerulonephritis is also present in pSS patients, classified as membranoproliferative glomerulonephritis. Cryoglobulinemic vasculitis, characterized by the deposition of immune complexes, is one of the most severe renal manifestations in pSS [164]. Overall, among pSS with renal involvement, the loss of renal function was reported in only 5–10% and may progress to end-stage kidney disease [168].

#### 3.4.1. Pathophysiology

Secondary to systemic inflammation, the infiltration of B and T lymphocytes and plasma cells into the renal interstitium was observed [169]. Interstitial fibrosis occurs as a result of tubulitis as well as the local production of autoantibodies against sodium chloride co-transporter and carbonic anhydrase in the collecting duct, which results in tubular dysfunction and renal tubular acidosis [170]. In glomerular involvement, an immune complex-mediated mesangioproliferative glomerulonephritis is present [171].

#### 3.4.2. Diagnosis

The diagnosis is a compound of clinical symptoms, such as edema in nephrotic syndrome, plus routine analyses with low proteinuria, elevated serum creatinine, and metabolic acidosis [164,168].

#### 3.4.3. Treatment

The prognosis of kidney disease associated with pSS is generally favorable. However, the treatment is very much dependent upon disease progression [164]. For the most common renal manifestations, especially in severe or active interstitial nephritis, treatment with systemic corticosteroids is recommended [172]. During disease relapse, a steroid-sparing agent is required, and AZA [172] or MMF [173] have been used with success. In type I distal RTA, alkaline products are prescribed for acidemia, while in persistent hypokalemia, supplemental potassium is required [171]. In membranoproliferative glomerulonephritis, and cryoglobulinemic vasculitis, specific treatment with immunosuppressants is recommended [174]. In severe cases, plasma exchange may be an option [171].

### 3.5. Hematologic Manifestations

In pSS, the hematologic manifestations can be divided into cellular and humoral components (Table 4). The cytopenias are the main cellular abnormalities, while hyper- and hypogammaglobulinemia, monoclonal gammopathy, cryoglobulinemia, and the presence of autoantibodies are the most frequent humoral manifestations [175]. Leukopenia was reported in 19% to 22% of pSS patients [176], while neutropenia and lymphopenia were noted in 14 to 27.3% [177] and in 14 to 23.9% of the pSS patients, respectively [116,178,179]. Secondary to chronic inflammation, anemia may occur in between 17.1 and 23.9% of patients [180], while thrombocytopenia was reported between 29.3 to 30.5% of pSS patients [181,182]. Hypergammaglobulinemia occur in 41.8% [183], while hypogammaglobulinemie is very rare [184]. Monoclonal gammopathy has been observed in few cases of pSS patients [185]. Low levels of C3 have been reported in 10–15% of patients [185], while low levels of C4 were detected in 5–20% [111,185].

Lymphoma is considered the most severe complications in pSS patients [184]. The most common type of lymphoma associated with pSS is the low-grade B cell non-Hodgkin lymphoma (NHL) of the marginal zone histologic type, especially that of mucosa-associated lymphoid tissue (MALT) [186].

**Table 4:** The prevalence of the main hematologic manifestations.

Hematologic Manifestations	% pSS Patients
Cellular manifestations	
Anemia	17.1–23.9% [177,187]
Leukopenia	19–22% [176,179,187]
Thrombocytopenia	29.3–30.5% [118,182]
Neutropenia	14–27.3% [118,177]
Lymphopenia	14–23.9% [118,178]
Humoral manifestations	
Hypergammaglobulinemia	41.8% [183]
Hypocomplementemia C3/C4	10–15%/5–20% [185]
Lymphoma	2.7–9.9% [184]

#### 3.5.1. Pathophysiology

Various environmental factors, such as viral infections, induce lymphocyte activation and their migration to target tissues [188]. In the process of lymphocyte migration and infiltration, chemokine receptors and their ligands play an important role [189]. The CC-chemokine receptor 7 (CCR7) has been recognized as a fundamental regulator directing lymphocytes to inflammatory lesions [181,190]. BAFF has been involved in the pathogenesis of pSS due to its role in B-cell regulation and proliferation [43]. Mutations of the BAFF receptor (BAFF-R) confer a higher risk for lymphoproliferation through the activation of the Nuclear Factor-κB (NF-κB) signaling pathways and of PI3K signaling pathways, and by inhibiting additive apoptotic pathways in pSS patients [46,191]. These interactions can play an important role in lymphoma progression. Seronegative pSS patients have less hyperactive B cells and thus a low risk for lymphoma development [192].

#### 3.5.2. Diagnostic

The diagnostic for hematologic manifestations is primarily biological. Additionally, in lymphoma, the clinical aspect is suggestive, with persistently swollen parotid glands secondary to autoimmune inflammatory sialadenitis, infection, and obstruction [193]. NHL manifests typically as a unilateral, persistent, and sometimes indurated nodule [184]. Ultrasound and MRI scans are helpful in the diagnosis and evaluation, while biopsy is mandatory for the final diagnosis [194]. The prognosis in NHL associated with pSS is usually good, especially with the MALT subtype [148]. The most frequently reported predictive factors in NHL include parotid enlargement, lymphadenopathy, palpable purpura, low C4 level, and cryoglobulinemia [35,176]. Elevated levels for rheumatoid factor, a focus score greater than three, and the detection of germinal centers in salivary biopsies are also highly predictive for lymphoma in pSS [195,196].

#### 3.5.3. Treatment

The treatment for hematologic involvement includes GCs, immunosuppressants, and biologic therapy [197,198]. Given the key role of BAFF in B cell clonal expansion and lymphoma development [195], it seemed that targeting BAFF and CD20 simultaneously through belimumab/rituximab co-administration could represent a promising therapeutic approach in for MALT subtype of pSS [92]. For patients with disseminated MALT, a personalized treatment should be considered, applying a B cell depletion strategy, that can be associated with chemotherapy [6,199] (Table 1). Alkylating agents such as CYC, doxorubicin, vincristine plus prednisone (R-CHOP), chlorambucil [176], the purine analog cladribine (2-cdA) [200], or fludarabine, can also be associated with standard chemotherapy [176]. Anti-TNF alpha therapy has been associated with an increased risk of lymphoma development in pSS patients. The reported mean time between initiation of therapy and the onset of the first symptoms of lymphoma was 23 months [201].

### 3.6. Pulmonary Manifestations

Pulmonary manifestations occur in up to 20% of pSS patients [202,203]. The most frequently reported pulmonary manifestations are airway disease [204], interstitial lung disease (ILD), and xerotrachea [111]. Upper airway dryness can promote chronic non-productive cough, nasal crusting, epistaxis, rhinosinusitis, and hoarseness in pSS patients [205]. Persistent chronic dryness predisposes to atelectasis, bronchiectasis, bronchiolitis, and recurrent episodes of respiratory tract infections [206]. Therefore, chronic cough is reported in up to 60% of pSS patients with pulmonary involvement [206]. The most common respiratory complication of pulmonary involvement is ILD, with a prevalence between 6 to 70% [207]. ILD can manifest as non-specific interstitial pneumonia (NSIP), with a prevalence between 29 to 42%, followed by lymphocytic interstitial pneumonia (LIP) between 4 to 15% and organizing pneumonia (OP) [208], while 11 to 43% of ILD patients may develop the usual interstitial pneumonia (UIP) [203,209]. Lymphoma prevalence in pSS is estimated to be between 1–2%, represented by low-grade extranodal marginal B-cell lymphoma of the MALT type [204].

Other rare complications in pSS patients with pulmonary involvement are amyloidosis, thromboembolic disease, and pulmonary arterial hypertension (Table 5) [204].

#### 3.6.1. Pathophysiology

A complex interaction of genetic environmental, and hormonal factors has been incriminated in pulmonary manifestations pathogenesis. Coughing symptoms are secondary to dryness from exocrine gland dysfunction and lymphocytic infiltration of the trachea, bronchi, and bronchioles [206,211]. In pSS patients, an increased formation of autoantibodies against the M3 R muscarinic receptor may lead to a compensatory increase in M3R expression, which finally leads to cholinergic hyperresponsiveness [212]. Human T lymphotropic virus type I (HTLV-1) seems to be one of the pathogens involved in the occurrence of pulmonary manifestations in pSS [213].

#### 3.6.2. Diagnostic

All asymptomatic patients should perform chest X-rays and pulmonary function tests (PFTs) every 6 to 12 months, while in those with symptoms, a bronchoalveolar lavage (BAL) and high-resolution CT scan (HRCT) is recommended [214,215]. In ILD, a restrictive pattern is typically noted on PFTs with a diminished diffusing capacity of carbon monoxide [204,216]. On HRCT, the NSIP presents typically as a symmetrical involvement with reticular changes, traction bronchiectasis, and ground glass opacities [204,217]. In LIP, the HRCT pattern describes nodules, ground-glass opacities, thickening of the interlobular septa, and cysts [218]. Additionally, the histopathology of LIP consists of diffuse polyclonal lymphocytic interstitial infiltrate with lymphoid follicles and germinal centers [219]. OP is typically represented by multiple areas of consolidation in the periphery with ground glass opacities and centrilobular nodules [215]. On biopsy samples, chronic inflammation and polypoid intraluminal masses of fibroblasts, myofibroblasts, and collagen in the alveolar ducts and adjacent spaces are observed [220]. In UIP, the HRCT pattern is characterized by reticular changes, bronchiectasis, and honeycombing at the bases and periphery [221,222]. Histopathology reveals minimal interstitial inflammation and patches of interstitial fibrosis [223,224]. In pSS patients with consolidating nodules, mass-like opacities, and mediastinal adenopathy, a lung biopsy must be performed to exclude lymphoma or other malignancies [204,215,225]. A systemic screening of all patients with pulmonary involvement is recommended according to the latest consensus in pSS management [225].

Predictive factors for ILD development in pSS patients include dry cough, dyspnea, Raynaud’s phenomenon, and anti-Ro52 antibodies [218]. Lower levels of forced vital capacity (FVC) and higher levels of serum Krebs von den Lungen-6 (KL-6) are predictive factors for worse prognosis in these patients [226].

#### 3.6.3. Treatment

Oral corticosteroids are the first-line therapy in symptomatic patients with progressive pulmonary impairment [204]. Furthermore, AZA or MMF, as steroid-sparing agents proved efficacy in ILD [224]. CYC combined with prednisone was successfully used in NSIP, OP, and combined patterns [208]. In patients with ILD following treatment with intravenous rituximab improvement was reported [227,228]. In a previous study on patients with LIP, a possible synergy of tacrolimus and a selective T-cell costimulatory inhibitor (CTLA4-Ig), abatacept, encouraging results of multitarget therapy were reported [88,229]. Low-dose IL-2 showed beneficial effects for patients with pSS and associated pulmonary lesions [222] (Table 1).

### 3.7. Gastrointestinal Manifestations

A spectrum of gastrointestinal (GI) manifestations has been described in pSS (Table 6). Esophageal involvement is represented by dysphagia in 65%, while gastroesophageal reflux (GER) is present in 13 to 60% of pSS patients with gastrointestinal manifestations [230,231]. Chronic diarrhea has been described in up to 9% of pSS patients and represents a diagnostic and therapeutic challenge [231,232]. Severe abdominal pain, GI bleeding, bowel infarction, or perforation are other manifestations reported in pSS patients [233]. Celiac disease associated with pSS was found with a prevalence between 4.5 to 15% [234,235,236]. Vasculitis involving the gastrointestinal tract in pSS is uncommon, and usually in association with cryoglobulinemia [119]. However, intestinal vasculitis should be considered in any pSS patient with severe abdominal pain, GI bleeding, infarcted bowel, or perforation [231]. Dysautonomia and chronic gastric inflammation with mucosal atrophy have also been described in pSS patients [3]. Therefore, autonomic dysfunction and gastroparesis were reported in 29 to 69% of pSS patients with gastric involvement [237,238]. In recent years, intestinal microbiota and its role in the pathogenesis of autoimmune diseases, including pSS, have been investigated [239,240].

Pancreatic involvement is typically asymptomatic and relates mostly to pancreatic exocrine insufficiency, with a prevalence of 36–63% [119,231]. Primary biliary cirrhosis (PBC), chronic active autoimmune hepatitis (AIH), and sclerosing cholangitis (SC) are liver manifestations in pSS [8,241]. PBC, the autoimmune disease of the bile ducts leading to bile duct destruction, cholestasis, and liver failure, was reported with a prevalence of 4% up to 9% [241], while AIH was confirmed in about 1 to 4% of pSS patients with liver involvement and was characterized by autoimmune destruction of hepatocytes and an increased serum level of autoantibodies and enzymes [242,243]. SC, characterized by progressive inflammation and fibrosis of the intra- and extra-hepatic bile ducts, is an exceptional manifestation in pSS patients [242].

**Table 6:** The prevalence of the main gastrointestinal manifestations.

Gastrointestinal Manifestations	% pSS Patients
Esophagus	
Dysphagia	65% * [231]
Gastroesophageal Reflux	13–60% * [231]
Stomach	
Gastritis	36–65% ** [244]
Gastrointestinal motility disfunction	29–69% ** [237]
Pancreas	36–63% [231]
Liver	49% [235]
Autoimmune hepatitis	1–4% *** [242,243]
Primary biliary cirrhosis	4–9% *** [243]
Small Intestine	
Chronic diarrhea	9% [231,232]
Celiac disease	4.5–15% [234,235]

* from patients with esophagus involvement. ** from patients with gastric involvement. *** from patients with hepatic involvement.

#### 3.7.1. Pathophysiology

The acid-neutralizing capacity of saliva is diminished due to decreased volume and altered pH [245]. Additionally, gastric acid production is inhibited due to a decrease in epidermal growth factor secretion from the submandibular glands in pSS patients [246]. The dysmotility in pSS patients is attributed to autoantibody activity that may inhibit muscarinic receptor-mediated cholinergic neurotransmission [247].

#### 3.7.2. Diagnostic

For the diagnosis of gastrointestinal involvement various paraclinical investigations might be used, such as endoscopy, barium swallow X-ray, or esophageal manometry [248,249]. The biopsy is indicated in atrophic gastritis, showing mononuclear cell infiltration of the mucosa and glandular atrophy with varying degrees of intestinal metaplasia [235]. Celiac disease also requires histopathologic confirmation [234]. Liver manifestations are diagnosed by laboratory parameters, ultrasonography, and in some cases, by biopsy. The prognosis of PBC is generally good, while the AIH can progress to cirrhosis and even hepatocellular carcinoma (HCC) [242,250].

#### 3.7.3. Treatment

In mild GI involvement, simple treatment strategies with secretagogue medication can improve the symptoms [88]. In more severe conditions, such as celiac disease or PBC, specific treatment is required [251]. In patients with PBC associated with pSS, the early use of ursodeoxycholic acid could prevent progression to cirrhosis [252]. In autoimmune hepatitis, treatment with prednisone followed by AZA was reported to be efficient [252], whereas in SC endoscopic interventions even liver transplant can be considered [242,253].

### 3.8. Cardiovascular Manifestations

Cardiovascular manifestations are rarely reported in pSS and are the main elements of the organ-specific group of non-ESSDAI features [3]. Cardiovascular events in pSS patients can be classified according to the interconnection between the traditional risk factors, glandular involvement [234,254,255,256] and the association with extra-glandular disease activity and longer disease duration [254]. A higher risk for major cardiovascular events, cerebrovascular events, and coronary events have been reported in pSS patients [257,258,259]. Furthermore, a higher prevalence of valvular regurgitation, systolic dysfunction, pericardial effusion, and lower coronary reserve have been diagnosed in pSS patients [260,261]. Similarly, an increased prevalence of pulmonary arterial hypertension (PH), reaching 12.5%, has been reported in pSS patients [262,263]. A clinical study has found that pSS patients with positive Ro/SS-A and La/SS-B antibodies had a higher prevalence of cerebrovascular events [264]. Antiphospholipid antibodies are found more frequently in pSS patients than in the general population [265]. However, only one-third of these patients will develop the antiphospholipid syndrome [266]. In pregnant women with positive Ro/SS-A antibodies, especially the anti-Ro 52 antibodies, an increased risk of developing autoimmune congenital atrioventricular block (CAVB) in the fetus has been reported. The first-born child has a risk for CAVB between 2 and 5%, that may increase up to 12% if the woman had a previous child with CAVB [267].

#### 3.8.1. Pathophysiology

The mechanisms for cardiovascular involvement remain unknown. Various studies have identified endothelial dysfunction, carotid intima-media thickness changes with resulting loss in vessel wall compliance in pSS patients [260,268]. Persistent endothelial dysfunction-related subclinical atherosclerosis may be found in pSS patients, who have increased values of circulating endothelial microparticles, endothelial progenitor cells [257], and angiotensin [269]. Endothelial dysfunction is considered one of the earliest changes that characterize atherosclerosis and seems to be more frequent in patients with higher disease activity scores [139]. Furthermore, BMPR2 mutation can cause the proliferation of pulmonary vascular smooth muscle cells, leading to PH [270]. The 5-HT4 serotoninergic receptor, the α1C and the α1D subunits of the L-type calcium channel, and the T-type calcium channel are in vitro fetus proteins that may be cross-reactive targets for anti-Ro 52 antibodies of women with pSS [271]. Thus, the arrhythmogenic effects of anti-Ro52 antibodies and their direct effect on fetus cardiocyte function were correlated with the inhibition of membrane calcium channels [272].

#### 3.8.2. Diagnostic

Potential biomarkers evaluating cardiac involvement and disease severity, such as C-reactive protein (CRP), IL-6, calprotectin, and dickkopf-related protein 1 (DKK-1), can be assessed in pSS patients [273]. Creatine kinase-MB, cardiac troponin I and T, brain natriuretic peptide, and pro-brain natriuretic peptide may also be used for monitoring cardiovascular involvement in pSS patients [273,274]. For long-QT syndrome diagnosis, ECG should be performed, while echocardiography with Tissue Doppler imaging and speckle-tracking technique can detect subclinical myocardial alterations [275]. When PH is suspected, right heart catheterization is recommended, and the diagnosis is defined as a pulmonary artery pressure above 25 mmHg [276]. Cardiac CT is recommended when pericardial involvement is suspected [255], while cardiac magnetic resonance (CMR) imaging [277] with tissue characterization sequences and positron emission tomography (PET) [278] provides additional insight regarding the presence of non-ischemic inflammatory myocardial involvement [279]. In recent years, CMR feature tracking (CMR-FT) has been used for the assessment of myocardial deformation with ventricular strain [277]. Overall, a high risk of cardiovascular events in pSS patients has been reported with longer disease progression and higher disease activity, with active immunologic and clinical features [262].

#### 3.8.3. Treatment

pSS patients can be considered at risk for cardiovascular diseases due to the systemic inflammation and immune dysregulation [258,280]. General preventive measures for cardiovascular manifestations represent the main treatment strategy in pSS patients as well. Having no specific recommendations for pSS patients with cardiovascular involvement, individualized treatment for each condition is recommended, according to the existing international guidelines [281].

### 3.9. Other Constitutional Symptoms

Fatigue is a challenge from both diagnostic and therapeutic perspectives in pSS patients. It is a widespread manifestation in pSS, alongside dry eye and dry mouth symptoms, being reported in up to 70% of patients [282]. Sleep disorders, dry mouth, anxiety, depression, fibromyalgia, anemia, or hypothyroidism have been incriminated in the pathogenesis of chronic fatigue [283,284]. Studies have shown that cytokines and other markers of inflammation are associated with fatigue in pSS [282,285]. Part of middle-aged women diagnosed with pSS may present a clinical triad characterized by dryness, pain, and fatigue [286]. Fatigue in pSS tends to persist over time, and regular physical exercise may prove beneficial [287]. HCQ has been mentioned in previous reports as a possible treatment option for fatigue, but there is currently no evidence of significant improvement [288,289]. Rituximab use for fatigue alleviation remains controversial [95,103]. In recent studies, low-dose IL-2 therapy has improved fatigue symptoms in pSS patients [222,290].

## 4. Future Perspectives for pSS Management

Genetic, proteomic and transcriptomic analyses are of interest in the characterization of the molecular and clinical variabilities among pSS patients. Analysis of the peripheral blood mononuclear cells (PBMCs) from pSS patients by single-cell RNA-sequencing (scRNA-seq) identified CD14+ monocytes (Mos), CD14+CD16+ Mos, CD16+ Mos, CD8+ CTLs, and CD56 CD16+FCER1G+ NK cells as the main players in pSS pathogenesis [291]. The weighted gene co-expression network analysis (WGCNA) indicated LINC00487 and SOX4 as key genes associated with the dysregulation of B cells in pSS patients [66]. Combining WGCNA and scRNA-seq, the ICOS gene up-regulation was found in salivary glands and PBMCs of pSS patients [292]. It was suggested that ICOS gene expression may be associated with lymphocytic infiltration in pSS patients and may become a useful biomarker for the detection of pSS and its complications [293]. Another study has detected an upregulation of Interferon induced with helicase c domain 1 (IFIH1) key gene in peripheral blood samples from mice and patients with pSS [294]. IFIH1 has been considered as a new diagnostic biomarker and potential therapeutic target in pSS patients. However, the clinical relevance of genetic testing in patients with pSS is still scarce, and the association of different gene variants with specific glandular and EGMs needs further investigation.

Proteomic studies have identified the upregulation of several salivary proteins, including neutrophil elastase, tripartite motif-containing protein 29 (TRIM29), calreticulin, clusterin, salivary NGAL, siglec-5, CA-VI, and vitronectin in pSS patients [295,296]. These unregulated proteins may help monitor the disease activity and predict the response to therapy in pSS patients. Furthermore, the TRIM29 protein might become an important marker due to its high diagnostic accuracy, particularly in pSS patients with anti-SSA/Ro antibodies [295].

Recent genome-wide transcriptome studies of salivary glands from mice observed that marginal zone B (MZB) cells are recruited during the early stage of the disease [297,298]. Blocking the lymphotoxin activity required for MZB cell ontogeny may prevent lymphomagenesis in pSS with EGMs [299]. Type 2 conventional dendritic cells (cDC2s) from patients with pSS are transcriptionally altered, inducing increased chemokine receptor CXCR5 expression and proliferation of tissue homing CD4+ T cells in pSS salivary glands [300]. Further investigation of cDC2s pathway in pSS may lead to future, more efficient therapies for patients [301].

The ongoing clinical trials investigating the benefits of biological therapies in pSS patients currently provide only preliminary but at the same time promising results that could lead to a radical shift in the overall management of this disease.

## 5. Conclusions

Diagnostic and therapeutic management in pSS, especially when extraglandular involvement is associated, is a major challenge for the clinician. It is very unlikely that a single therapy will provide satisfying results or long-term disease control in pSS patients with EGMs, given the heterogeneity of clinical and biological phenotypes. Therefore, a better knowledge of the pathogenesis and biological profile for each type of EGM associated with pSS is mandatory. Continued research in the pathogenic mechanisms and biomarkers field are necessary for the timely detection of EGMs in pSS patients, thus preventing serious complications. Biological technologies such as genetic, proteomic, or transcriptomic analysis could lead to updated, highly accurate diagnostic guidelines in pSS and could become the fundament for future personalized therapies in the management of this disease.

## Figures and Tables

**Table 1:** Clinical trials that investigated the effects of biological therapies in pSS.

Therapy	References Year	Study Characteristics			Outcome
		Type/Phase	Cohort Size (*n* =)	Follow-Up Period (w)	
Rituximab	Carubbi et al., 2013 [91]	RCT	41	120	↓ ESSDAI activity, ↓ glandular infiltrate, ↓ ectopic GC
	Devauchelle- Pensec et al., 2014 [94]	RCT	120	24	Fatigue alleviation
	Cornec et al., 2016 [47]	Open label	45	24	Mild glandular B-cell depletion
	Bowman et al., 2017 [95]	RCT/III	133	26	No clinical efficacy
	Fisher et al., 2018 [96]	RCT/III	52	48	Improvement in ultrasound score
Belimumab	Mariette et al., 2013 [97]	Open label/II	30	28	Reduction in parotid swelling, Mild ↓ of B cell activation
	de Vita et al., 2015 [98]	Open label/II	30	52	Improvement in ESSDAI score
	Quartuccio et al., 2016 [99]	Open label/II	13	52	No significant changes in type II IFN scores
Belimumab/Rituximab	Mariette et al., 2022 [100]	RCT/II	86	68	B cell depletion in salivary gland
Abatacept	Adler et al., 2013 [101]	Open label	11	24	↓ glandular inflammation, ↑ saliva production
	Haacke et al., 2017 [102]	Pilot RCT	15	24	Inhibition of local formation of memory B-cells
	Verstappen et al., 2017 [103]	Open label	15	48	↓ cTfh-cells and expression levels of the activation marker ICOS on T-cells
	Baer et al., 2020 [104]	RCT/III	187	24	No significant clinical efficacy
	de Wolff et al., 2022 [105]	RCT/III	40	48	Improvement in ESSDAI activity and eyes dryness.
Tocilizumab	Felten et al., 2020 [106]	RCT	110	44	No significant clinical efficacy
Ianalumab (VAY736)	Bowman et al., 2022 [107]	RCT/IIb	190	24	↓ ESSDAI activity
	Diekhoff et al., 2022 [108]	RCT	27	24	Improvement in salivary gland ultrasound score
LD-IL-2	He et al., 2022 [109]	RCT/II	60	12	Restore the balance of T and B cell subsets

Abbreviations: RCT, randomized control trial; ↓, low; ↑, high; ESSDAI, EULAR Sjögren’s syndrome disease activity index; GC, germinal centers; IFN, interferon; cTfh cells, circulating T follicular helper cells; ICOS, inducible costimulator; LD-IL-2, low-dose-interleukin-2.

**Table 5:** The prevalence of the main pulmonary manifestations.

Pulmonary Manifestations	% pSS Patients
Airway disease	
Cough—Xerotrachea	41–61% [206]
Bronchiectasis	7–54% [206,210]
Interstitial lung disease	6–70% [207,210]
Non- specific interstitial pneumonia (NSIP)	29–42% * [208,209]
Usual interstitial pneumonia (UIP)	11–43% * [203,209]
Lymphocytic interstitial pneumonia (LIP)	4–15% * [111,208]
Organizing pneumonia (OP)	9.5% ** [208]
Others	
MALT lymphoma	1–2% [204]

* from patients with airway disease. ** from interstitial lung disease.

## Data Availability

Not applicable.
